# Special Issue “Valorization of Lignocellulosic Biomass”

**DOI:** 10.3390/ijms27020932

**Published:** 2026-01-17

**Authors:** Haishun Du, Sonali Mohapatra, Xuejun Pan

**Affiliations:** 1 Department of Biological Systems Engineering, University of Wisconsin-Madison, Madison, WI 53706, USA; 2 Department of Forestry, Michigan State University, East Lansing, MI 48824, USA

The sustainable valorization of lignocellulosic biomass continues to attract significant scientific and technological interest as societies seek renewable alternatives to fossil-based materials, chemicals, and fuels [1,2,3]. Owing to its structural diversity and chemical richness, lignocellulosic biomass offers numerous pathways for transformation, ranging from the development of high-performance functional materials to the selective depolymerization of aromatic polymers and the biological or thermochemical conversion of polysaccharide-rich feedstocks [4,5]. Unlocking this potential, however, requires advances across multiple research fronts, including molecular-level understanding, catalyst and enzyme design, microbial regulation, and process engineering strategies that enable scalability and environmental compatibility [6,7].

This Special Issue brings together nine contributions that collectively showcase the breadth of research in this area and illustrate how complementary approaches can accelerate the development of integrated, circular biorefinery systems. The studies span four thematic areas. The first explores how the intrinsic architectures of lignocellulosic and biological resources can be leveraged to create functional materials for energy harvesting, elastomer reinforcement, and biomedical applications. The second focuses on lignin structural chemistry and emerging depolymerization strategies that enable the controlled production of value-added aromatic intermediates. The third examines enzymatic and microbial mechanisms underpinning hemicellulose and lignocellulose deconstruction, providing insight into synergistic enzyme action and regulatory proteins that shape fungal degradation programs. The fourth highlights thermochemical and biochemical conversion routes, including hydrothermal carbonization for environmental remediation and fed-batch high-solids hydrolysis for producing fermentable sugars and lignocellulosic ethanol.

Together, these contributions underscore both the complexity and the opportunity inherent in biomass utilization. They demonstrate that advancing sustainable bioproducts increasingly depends on the integration of materials science, chemistry, enzymology, microbiology, and process engineering. By bridging these complementary disciplines, the studies in this Special Issue deepen our understanding of biomass conversion and expand the spectrum of pathways for producing renewable materials, chemicals, and fuels.

## 1. Biomass-Derived Functional Materials

Recent advances in lignocellulosic biomass utilization reflect a growing shift toward designing functional materials that preserve and exploit the intrinsic architecture of lignocellulosic and biological resources [8,9]. Rather than fully deconstructing biomass into platform chemicals, emerging strategies aim to harnessing its native morphology, crystallinity, anisotropy, and chemical functionalities to develop high-performance materials for energy, electronic, elastomeric, and biomedical applications [10,11,12]. This Special Issue features three studies demonstrating how diverse biomass-derived structures can be transformed into functional materials for energy harvesting, elastomer reinforcement, and biomedical protection.

### 1.1. Lignocellulose for Green Energy Harvesting and Electronics

Troncoso and coworkers present a comprehensive review of lignocellulose-based triboelectric nanogenerators, highlighting how wood, cellulose fibers, nanocellulose, and chemically modified lignocellulosic substrates offer favorable triboelectric properties, tailored surface morphology, and mechanical robustness. The review outlines fabrication methods, device architectures, and emerging applications in wearable health monitoring, environmental sensing, and smart packaging. It also addresses key challenges such as environmental stability and performance optimization, providing valuable guidance for the future development of eco-friendly and self-powered electronic systems (Contribution 1).

### 1.2. Rod-like Cellulose Reinforcement in Natural Rubber

Yuan and collaborators report a bottom-up strategy for reinforcing natural rubber through the in situ regeneration of rod-like cellulose nanostructures within the rubber matrix. Microcrystalline cellulose is first dissolved in a NaOH/urea aqueous system at −18 °C, blended with natural rubber latex, and then regenerated using 5 wt% acetic acid, which precisely controls precipitation and promotes the formation of uniformly dispersed rod-like regenerated cellulose (RRC). Under deformation, these RRC units rapidly assemble into rigid filler networks, delivering a pronounced reinforcement effect and an earlier stress upturn compared with neat rubber. By avoiding the aggregation issues common in top-down nanocellulose approaches, this method provides a scalable route to high-aspect-ratio cellulose fillers and highlights the value of controlled self-assembly for sustainable and high-performance elastomers (Contribution 2).

### 1.3. Upcycling Cellulose-Rich Fungal Residues into Bioactive Hydrogels

Although fungal biomass is not strictly lignocellulosic, its cell-wall polysaccharides, composed of glucans, chitin, and bioactive compounds, represent a valuable renewable resource for materials development. Huang’s group demonstrated a closed-loop upcycling approach by converting extraction residues of *Antrodia cinnamomea* into glucan-rich hydrogels that retain bioactive compounds, including triterpenoids and antioxidants. These hydrogels exhibit strong antibacterial activity against *Staphylococcus aureus* and effectively protect HaCaT keratinocytes from UVB-induced damage. This study illustrates how fermentation residues can be valorized into multifunctional hydrogels for wound care and skin protection, further emphasizing the potential of biomass resources in biomedical applications (Contribution 3).

## 2. Lignin Structural Chemistry, Depolymerization, and Emerging Valorization Pathways

As the only large-volume renewable aromatic biopolymer, lignin represents both a long-standing challenge and an exceptional opportunity for sustainable chemical and material innovation [13]. Its heterogeneous aromatic network, dominated by β–*O*–4 ether linkages but intertwined with extensive C–C bonding and diverse functional groups, demands a deeper molecular-level understanding and the development of gentle, selective depolymerization strategies [14,15]. The two contributions in this Special Issue provide complementary advances: one elucidates the solid-state molecular organization of lignin-derived compounds, and the other establishes a scalable, green depolymerization route. Together, these studies offer a clearer picture of how lignin can be efficiently converted into high-value chemicals and materials.

### 2.1. Solid-State Features and Molecular Packing in Lignin Hydrogenolysis Products

Driscoll and coworkers deliver an insightful analysis of the solid-state structures of monomers and dimers prevalent in lignin hydrogenolysis oils, using single-crystal X-ray diffraction supported by DSC and intermolecular energy calculations. Their study reveals how hydrogen bonding, π–π stacking, and C–H···π interactions govern the packing of key molecules such as dihydroconiferyl alcohol, propyl guaiacol glycidyl ether, and eugenol glycidyl ether. Notably, glycidylated monomers form unexpectedly ordered π–π stacked structures despite lacking classical H-bond donors. These findings help explain selective crystallization, phase-separation behavior, and thermal properties observed in lignin-derived oils, offering molecular-level guidance for designing epoxy precursors, tuning polyol behavior, and improving downstream separation in lignin valorization (Contribution 4).

### 2.2. Mild and Scalable Oxidative Depolymerization Toward Functional Lignin Fractions

Lourenço and collaborators demonstrate a solvent-free, catalyst-free oxidative depolymerization strategy that converts kraft and LignoBoost lignins into low-molecular-weight, oxygen-rich aromatic fractions using only hydrogen peroxide at mild conditions. The internally generated acidic environment efficiently cleaves β–*O*–4 linkages and oxidizes side chains, yielding aromatic dimers/trimers and aliphatic dicarboxylic acids. This versatile method works across lignin types and remains effective upon 25-fold scale-up, producing narrowly dispersed, highly functionalized products that can be readily crosslinked into thermally stable biopolymers. This work highlights peroxide-driven oxidative cleavage as a practical and green route to functional lignin oligomers for sustainable polymer development (Contribution 5).

## 3. Enzymatic and Microbial Tools for Hemicellulose and Lignocellulose Deconstruction

Efficient biological deconstruction of lignocellulosic biomass relies not only on catalytic enzymes but also on regulatory proteins that coordinate fungal metabolic networks. Two studies in this Special Issue provide complementary insights: one examines the of synergistic action of hemicellulose-active enzymes, while the other explores small secreted regulatory proteins that shape fungal lignocellulolytic pathways.

### 3.1. Synergistic GH62 α-L-Arabinofuranosidases Enable Efficient Hemicellulose Deconstruction

Méndez-Líter and collaborators characterize two GH62 arabinofuranosidases from *Talaromyces amestolkiae*, revealing their strong cooperative action with xylanases in arabinoxylan degradation. Despite belonging to the same enzyme family, the two GH62s differ markedly in substrate tolerance, thermostability, and kinetic behavior, enabling them to target complementary positions on the arabinose-decorated xylan backbone. When combined with xylanases, they significantly enhance xylooligosaccharide release, underscoring the critical role of enzymatic synergy in hemicellulose remodeling. These findings support the rational formulation of hemicellulase cocktails for biofuel production and the tailored generation of prebiotic xylo-oligosaccharides (Contribution 6).

### 3.2. Small Secreted Proteins as Regulators of Fungal Lignocellulose Conversion

Yarden and coworkers provide a mechanistic perspective on how small secreted proteins (SSPs) regulate lignocellulose degradation in *Pleurotus ostreatus*. Transcriptomic analysis during growth on wheat straw identifies ssp4 and ssp6 as the predominant SSPs on lignocellulosic substrates. Silencing the ssp family alters the degradation program, reducing lignin breakdown through strong downregulation of key peroxidases (vp2 and mnp1) with upregulating hemicellulose- and pectin-related pathways. This adaptive transcriptional reorganization highlights SSPs as higher-level regulators of enzyme networks, substrate preference, and fungal physiology, positioning them as promising targets for engineering more efficient biomass-degrading fungi (Contribution 7).

## 4. Thermochemical and Biochemical Conversion Pathways for Biomass Valorization

In addition to advances in functional materials, lignin chemistry, and enzymatic/microbial deconstruction, scalable biomass valorization also requires robust process technologies capable of handling heterogeneous feedstocks under challenging environmental constraints. The two studies featured in this section address these needs from complementary angles. One demonstrates how hydrothermal carbonization can simultaneously remediate and convert heavy-metal-contaminated biomass, while the other develops an industrially relevant fed-batch strategy that overcomes the limitations of high-solids enzymatic hydrolysis. Together, these contributions highlight the critical role of thermochemical and biochemical engineering in enabling safe, efficient, and economically viable biorefinery systems.

### 4.1. Hydrothermal Carbonization as a Dual-Purpose Route for Biomass Remediation and Valorization

Wang and coworkers investigate hydrothermal carbonization (HTC) as an integrated strategy for treating heavy-metal-contaminated biomass while producing a value-added carbonaceous solid. Under HTC conditions, metals such as Cu, Zn, Cd, and Pb are efficiently immobilized within the hydrochar and converted into more stable inorganic forms. Tessier fractionation confirms a marked reduction in the exchangeable and carbonate-bound fractions, demonstrating significantly lower ecological risk and reduced potential for secondary pollution. By simultaneously stabilizing toxic metals and generating carbon-rich hydrochar, this work positions HTC as a practical dual-function technology that unifies environmental remediation with the safe valorization of contaminated residues (Contribution 8).

### 4.2. High-Solids Fed-Batch Hydrolysis for Industrially Relevant Sugar Platforms and Ethanol Production

Huang and collaborators develop a fed-batch high-solids enzymatic hydrolysis strategy that addresses key barriers in industrial biomass processing, particularly high viscosity, mass-transfer limitations, and enzyme inhibition at elevated solid loadings. By gradually increasing the solids content to 30%, the authors achieve hydrolysates containing 161–205 g/L fermentable sugars from corn stover and corn cob. Without requiring intermediate concentration steps, these hydrolysates support simultaneous saccharification and co-fermentation, producing ethanol titers of up to 72.8 g/L. The study provides practical engineering insights into managing rheology and inhibition effects in high-solids systems and demonstrates a scalable route toward economically competitive second-generation ethanol and sugar-platform biorefineries (Contribution 9).

## 5. Conclusions

The studies in this Special Issue highlight notable progress in lignocellulosic biomass valorization and point to several converging directions for future research. One emerging trend is the selective, structure-informed utilization of biomass, as demonstrated by advances in triboelectric materials, regenerated cellulose reinforcements, and bioactive hydrogels. Continued work in this area will benefit from deeper insights into hierarchical structure–property relationships and the development of scalable processing strategies.

Lignin-focused contributions underscore the importance of molecular-level understanding and mild depolymerization approaches. Clarifying solid-state interactions and developing solvent-free oxidative pathways together provide promising routes to controlled aromatic intermediates. Extending these strategies to diverse lignin streams and integrating them with downstream polymer development will be critical for broadening lignin’s technological relevance.

Biological deconstruction studies emphasize both catalytic synergy and regulatory control. Synergistic hemicellulases and small secreted proteins offer new opportunities for optimizing enzyme cocktails and engineering more efficient biomass-degrading systems. Future advances will rely on combining omics-enabled discovery, protein engineering, and systems-level metabolic regulation.

Thermochemical and biochemical process innovations further demonstrate the need for robust, scalable engineering solutions. Hydrothermal carbonization enables simultaneous remediation and conversion of contaminated biomass, while high-solids fed-batch hydrolysis addresses major bottlenecks in industrial saccharification and ethanol production. Continued integration of process design with techno-economic and life-cycle assessments will be essential for commercial translation.

Collectively, these contributions reflect the interdisciplinary nature of current biorefinery research and illustrate how progress across materials science, chemistry, biology, and engineering can drive the development of efficient and sustainable biomass utilization pathways.
